# Dorsal Perilunate Dislocation Coupled With a Distal Radius Fracture: A Report of a Rare Case

**DOI:** 10.7759/cureus.103218

**Published:** 2026-02-08

**Authors:** Samir Sakaria, Mariafe Reyes, Parth Chandan, Gary Schwartz

**Affiliations:** 1 Department of Medical Education, Nova Southeastern University Dr. Kiran C. Patel College of Allopathic Medicine, Fort Lauderdale, USA

**Keywords:** distal radius fracture, high-energy trauma, mechanism of injury, perilunate dislocation, wrist trauma

## Abstract

High-energy traumatic injuries to the wrist have been reported in the literature, which may result in isolated perilunate dislocations or distal radius fractures (DRFs). Dorsal perilunate dislocations are reported to be more common compared to volar dislocations. The concurrent presentation of a dorsal perilunate dislocation with a DRF has been rarely reported. Objective post-operative outcomes of this injury pattern, such as active motion, sensation, and function, are important with follow-up because of the complexity of this condition.

The purpose of this report is to present the clinical presentation, the radiographic evaluation, and the approach used in the diagnosis and treatment of this uncommon, combined injury.

## Introduction

Perilunate dislocations are associated with high-energy trauma, resulting in carpal disruption and malalignment [1]. Perilunate fracture-dislocations are uncommon injuries, representing approximately 10% of carpal injuries [2,3]. In a perilunate dislocation, there is collinearity between the lunate and the lunate fossa of the radius; however, the capitate and structures that lie distal to it have an abnormal articulation with the lunate. This disruption in collinearity can be observed as a dorsal perilunate dislocation or a volar perilunate dislocation, with the former occurring more frequently [4]. Distal radius fractures (DRFs) are a common injury, but complex fracture patterns can make fixation challenging [5]. DRFs account for approximately 17% of all fractures seen in the adult population in the emergency department in the United States. Due to the high incidence of DRFs, there are many non-surgical and surgical treatment options [6]. Surgical treatment interventions include: (i) closed reduction and percutaneous pinning with Kirschner wires, (ii) external fixation, (iii) open reduction internal fixation (ORIF) with volar or dorsal plate fixation (PF), and (iv) intramedullary nailing [7].

This case report details the presentation of a dorsal perilunate dislocation with a concurrent comminuted intra-articular fracture of the distal radius.

## Case presentation

The patient was a 32-year-old right-hand dominant man who worked as a window installer. He presented with pain in the right wrist following a motorcycle accident where he was struck by a car, fell, and landed on his dorsiflexed right wrist, suffering an axial load. He reported numbness in the thumb, index, long, and ring fingers. The patient is a 10-pack-year smoker. He was a social drinker of alcohol and did not use any illicit drugs. No other comorbidities were present.

On physical examination of the right upper extremity, there was no tenderness in the shoulder or elbow. There was tenderness and swelling on the dorsal and volar aspect of the wrist and hand as well as decreased digital motion. Two-point discrimination in the thumb, index, and long fingers was 12 mm, and in the ring and small fingers was 6 mm. He had difficulty with thumb opposition. There was no evidence of thenar atrophy. Thenar sensation was normal. There was a positive percussion test at the carpal tunnel and a negative percussion test at the Tunnel of Guyon.

**Figure 1:**
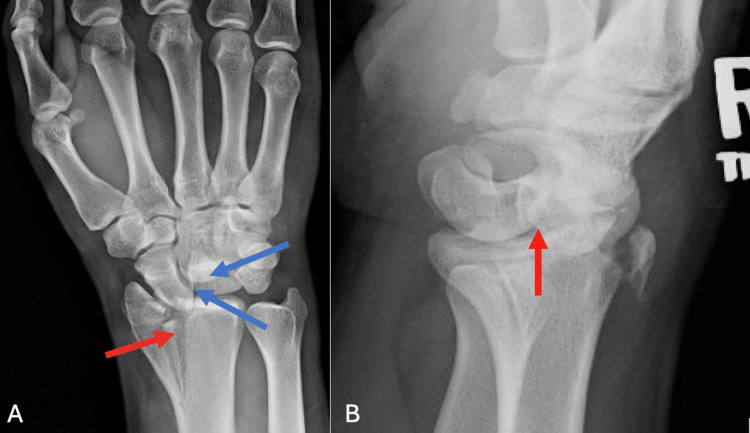
Preoperative Imaging Preoperative posterior-anterior view (A) and lateral view (B) of the right wrist demonstrate a comminuted intra-articular displaced fracture of the distal radius with a die-punch fragment along with a dorsal perilunate dislocation showing loss of collinearity between the lunate and the capitate (red arrows). The Gilula arcs are disrupted (blue arrows).

X-rays demonstrated a comminuted intra-articular fracture of the right distal radius with a die-punch fragment, as well as a dorsal perilunate dislocation. There was disruption of Gilula’s arcs on the anterior-posterior view and loss of collinearity between the lunate and capitate on the lateral view (Figure 1).

**Figure 2:**
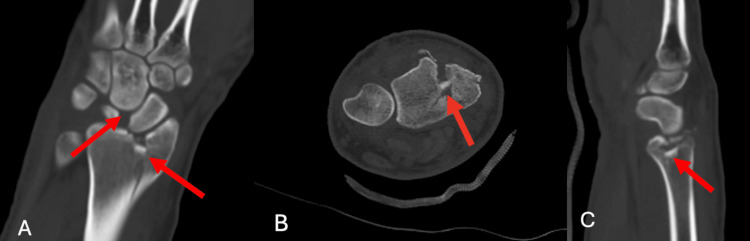
Preoperative Computerized Tomography of the Right Wrist Preoperative CT coronal (A), axial (B), and sagittal (C) imaging of the right wrist after a closed reduction demonstrates the comminuted fracture of the distal radius with a residual scapho-lunate diastasis. There is collinearity between the distal radius and carpal bones (red arrows).

A closed reduction of the dorsal perilunate dislocation of the right wrist was performed in order to decrease the pressure on the median nerve on the day of presentation. A CT scan after the closed reduction demonstrated the depressed cortical wall fragment of the distal radius and reduction of the perilunate dislocation (Figure 2).

**Figure 3:**
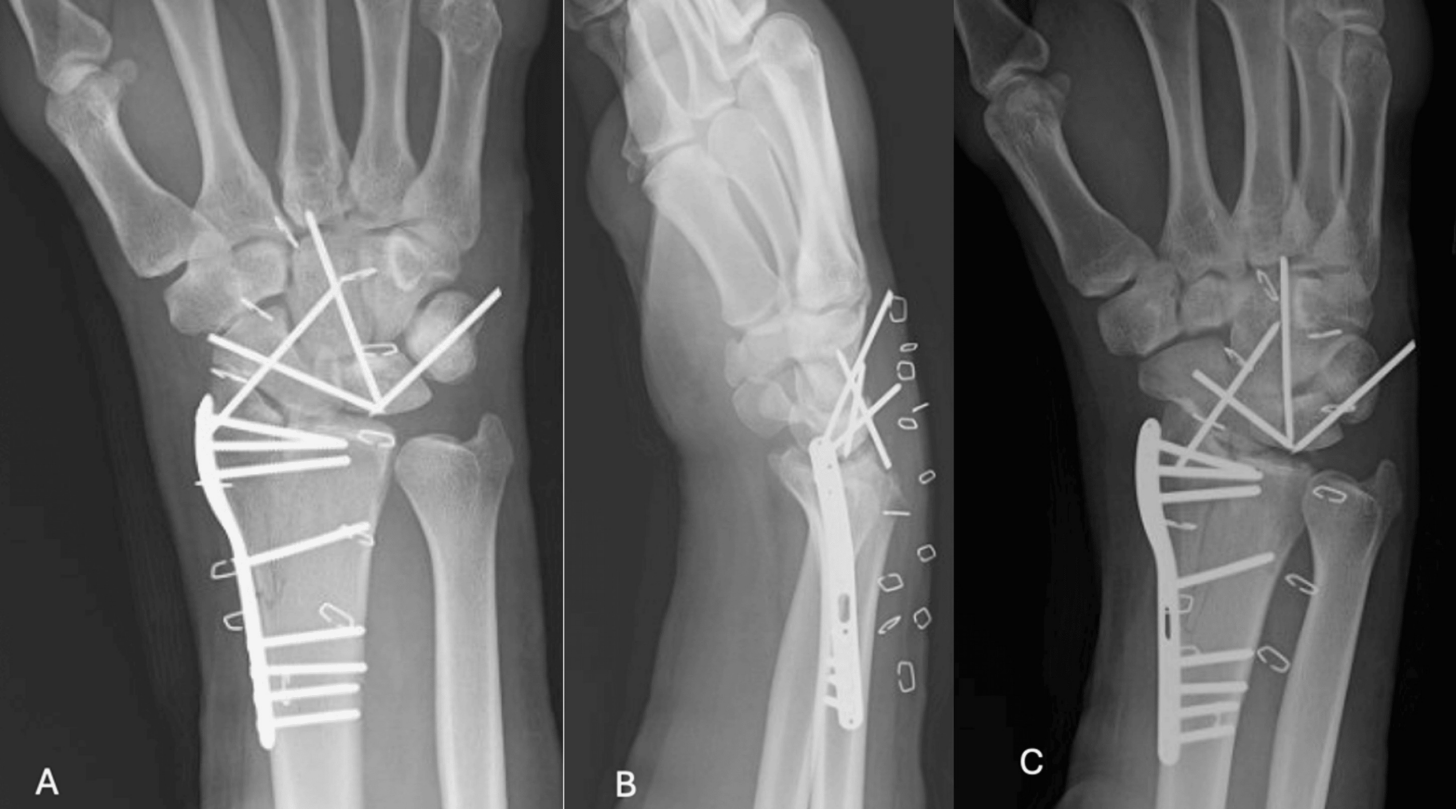
Postoperative Imaging of the Right Wrist Postoperative posterior-anterior (A), lateral (B), and oblique (C) imaging after fixation demonstrated fixation of the distal radius with a radial column plate and fixation of the carpus with Kirschner wires. There is collinearity between the distal radius and carpal bones and no residual scapho-lunate diastasis. Restoration of radial-carpal and inter-carpal alignment is present.

The patient also sustained a left foot and ankle fracture dislocation. The patient subsequently underwent ORIF of the right DRF and the right perilunate dislocation, right carpal tunnel release, and volar capsulorrhaphy of the right wrist the day after presentation (Figure 3). The intercarpal ligaments were damaged to the point that repair was not possible.

The surgery was performed under general anesthesia with endotracheal intubation with the patient in the supine position. A tourniquet was applied and was inflated for 120 minutes during the dissection portion of the procedure. A volar incision was utilized to initially approach the carpal tunnel with median nerve decompression, the volar capsule, as well as the radial column of the distal radius. There was a large rent in the volar capsule which was repaired.

The median nerve was noted to be contused but intact. A dorsal approach was performed to approach the die-punch fragment of the distal radius and the dorsal perilunate dislocation. Regarding the fixation of the perilunate dislocation, the lunate was initially transfixed to the distal radius with a .062-inch Kirschner wire. Subsequently, the scaphoid was transfixed to the lunate, the capitate to the lunate, and the triquetrum to the lunate. The Kirschner wire transfixing the lunate to the radius was then removed. There was excessive swelling to close the volar wound. Therefore, this wound was left open. Dressings were applied to keep the wound moist. Once a granulation base was present, a split-thickness skin-graft was placed one week later. In the immediate post-operative period, the sensation in the median nerve distribution improved slightly but totally resolved over the course of six weeks. Occupational therapy was prescribed for the patient but due to financial concerns, he did not comply with this recommendation. He, therefore, was working on a home-therapy program. This included active and passive motion of the digits, wrist, forearm, and elbow, edema control, and retrograde massage for the first two weeks. From weeks 3-5, the patient was to work on therapeutic activities such as buttoning, tying shoes, zipping, use of clothespins and the use of therapeutic putty. From the sixth week on, the patient was to work on strengthening exercises (Table 1).

**Table 1:** The Home Therapy Program Prescribed to the Patient

Weeks	Activity
1-2	Active & passive range of motion to the digits, wrist, forearm, and elbow, edema control, retrograde massage
3-6	Therapeutic activities such as buttoning, tying shoes, zipping, use of clothes pins, therapeutic putty, writing, and typing
>6	Strengthening exercises

The Kirschner wires were subsequently removed at eight weeks post-operatively. The patient continued with a home therapy program. He was lost to follow-up 12 weeks after the index operation. Due to this, no further follow-up radiographs were obtained. Further follow-up is important due to potential complications such as stiffness, degenerative joint disease, carpal instability, and avascular necrosis.

## Discussion

Dorsal perilunate dislocations and DRFs each can be the result of a high-energy traumatic injury [1]. The direction of force transmission can result in complex injuries involving multiple joints and ligaments, making DRFs with dislocations particularly challenging [5]. In our patient’s case, the uncommon occurrence of a dorsal perilunate dislocation along with a comminuted intra-articular fracture of the distal radius was diagnosed. Combined injuries, such as dorsal perilunate dislocations with DRFs, are rare but highly complex and often require meticulous surgical planning and management [8].

Similar combined injuries have been rarely reported in the literature. In 1967, Galasko reported one of the first cases demonstrating a combined presentation of a trans-radial dorsal perilunate fracture-dislocation of the wrist and fractured radial styloid [9]. In 2010, Hénault et al. detailed a similarly unusual presentation of a posterior perilunate carpal dislocation with an articular comminuted DRF [3]. At the time, this specific presentation had not been described, and the case report details a poor functional outcome due to severe wrist mobility limitations despite early surgical intervention. This underlines the importance of increasing awareness of the complexity and prognosis associated with dorsal perilunate dislocations with DRFs. These authors noted the rare occurrence of radial styloid fractures that present alongside perilunate fracture-dislocations [2]. These cases together demonstrate how, in combination, these two conditions are an unusual presentation. A retrospective study done over a 17-year period by Leung et al. found that among 131 patients with perilunate fracture/dislocations, and among the 22 different complex fracture patterns, only five patients had an associated DRF, making this combination especially rare [10]. 

Additional studies note the importance of the mechanism of injury eliciting the combined presentation of injuries previously discussed. Chen et al. described a case exhibiting an ulnar-sided force leading to the presentation of a dorsal and radial perilunate dislocation associated with a fracture through the radial styloid [2]. A study by Wu et al. emphasized the role of extensive dorsiflexion, axial loading, and twisting mechanisms leading to DRFs associated with lunate dislocation [11].

These mechanisms of injury appear to be relatively more common than others including injuries with forces applied to the ulnar side. A study by Enoki et al. detailed the more unusual mechanism of a force applied to the ulnar side of the wrist and hand, resulting in a distinct fracture-dislocation pattern [12]. These studies demonstrate how aspects such as the mechanism of injury can suggest variations in the specific presentation of the combined fracture and dislocation. 

Moreover, a study by Fowler described how ligament injuries, including scapholunate ligament and lunotriquetral interosseous ligament injuries, contribute to the co-occurrence of dorsal perilunate dislocations and DRFs. These ligament injuries were previously believed to be easily detected on radiographs based on the SL interval [13]. Additionally, a study by Richards et al. found that the SL interval was within two millimeters or less in 60% of cases where an SL tear was confirmed arthroscopically [14]. The findings from these two studies underline the importance of keeping a high index of suspicion if initial radiographs do not show significant SL diastasis since the SL diastasis may only become apparent after fracture reduction. A summary of the key findings, associated nerve injuries, treatment, and recovery time is seen in Table 2.

**Table 2:** A Summary of the Key Findings, Including the Type and Number of Fractures and Dislocations, Mechanism of Injury, Associated Nerve Injuries, Treatment, and Recovery Time

Reference (AMA format)	Type of Fracture (if Applicable)	Type of Dislocation (if Applicable)	# of Fractures/Dislocations Discussed in the Study	Mechanism of Injury	Nerve Injury (if Applicable)	Treatment Method (include Surgical Approach)	Recovery Time
Combined Presentation of Perilunate Dislocation and Distal Radius Fracture							
Hénault et al. (2010) [3]	Complex comminuted articular distal radius fracture	Dorsal perilunate dislocation	1 combined association of fx and dislocation	Fell onto a hyperextended wrist	Paresthesia in all fingers	ORIF w K-wires and fixation via osteosynthesis devices; carpal tunnel release	-2 yr follow-up “unsatisfactory functional results”; wrist flexion & extension limited to 5 degrees, wrist abduction 20 degrees, wrist adduction 5 degrees, wrist strength 12% of contralateral side; Cooney functional score of 40; no residual sensory complications
Enoki et al. (2008) [12]	Comminuted and displaced radial styloid fx; Radially displaced ulnar styloid fx; Sagittal oblique fracture through lunate; Avulsion fx at the ulnar pole of lunate	Radially displaced dislocation of the carpus	N/A	Fell onto wrist; Ulnar to radial direction force (most case reports discuss a radial to ulnar force)	Motor & sensory exam was normal (no clinical nerve injury found) but in OR hemorrhagic tenosynovium was noted, raising concern for subclinical injury.	Closed reduction immediately in ED; Followed by open reduction (volar approach) with K wires; Carpal tunnel release	K wires removed at 10 weeks; at nine months, radial styloid washer and screw removed. Arthroscopic exam of the wrist showed no rupture of the scapholunate ligament. No functional results discussed
Chen et al. (2013) [2]	Radial styloid fracture (case 1); Ulnar styloid fracture (case 1); Fracture of triquetrum (case 1); Lower extremity fxs (case 2)	Palmar and radial perilunate dislocation (case 1); Dorsal transstyloid perilunate dislocation (case 2)	N/A	MVA → right wrist pressed underneath rest of body (case 1); Fell onto wrist where it was pressed under the body (case 2)	N/A	Following immediate closed reduction, percutaneous reduction and fixation of the radial styloid. (case 1); No reduction or fixation of ulnar styloid or triquetrum (other than initial closed reduction - case 1); Closed reduction → dorsal approach ORIF	12-month follow-up, no complaints of wrist pain, 60-degree flexion, 40-degree extension, 30-degree ulnar deviation, 20-degree radial deviation (case 1); 12-month follow-up, mild pain after exertion, 75-degree flexion, 45-degree extension, 30-degree ulnar deviation, 25-degree radial deviation (case 2)
Wu et al. (2013) [11]	Distal radius fracture associated with lunate dislocation. Includes dorsal and radial displacement fractures, fractures of the radial styloid process, and cases of inversion or anterior dislocation of the lunate.	Lunate was anteriorly dislocated and rotated towards the palm by nearly 90°.	58 patients in total; 36 with acute distal radius fractures and lunate dislocation. 22 with old distal radius fractures and lunate dislocation (more than three weeks post-injury).	High-energy trauma: Falls from height, causing extreme dorsiflexion injuries (50 patients). -Twisting injuries (8 patients).	-Median nerve	Acute Injuries: Conservative Treatment: Manual reduction under local anesthesia. Plaster immobilization in a flexed position. Minimally invasive surgical treatment: Poking reduction through a small incision. Kirschner wires (K-wires) are used to fix the lunate and radius. External fixation with plaster splinting. Old Injuries: Open reduction and internal fixation. Surgical Approaches: Dorsal approach with repair of the dorsal radiolunate ligament. Palmar approach for reducing the lunate dislocation. Post-Surgery Fixation: Plaster immobilization for two weeks, followed by six weeks of external fixation.	Acute Injury: Excellent outcomes in 91.7% of patients with early treatment. Old Injury: Lower success rate (54.5%) and higher rates of lunate necrosis (27.2% overall). Lunate necrosis was more common with the palmar approach (50%) compared to the dorsal approach (14.3%).

The surgical management included an emphasis on the importance of restoring carpal alignment and maintaining joint stability through approaches like ORIF, combined with Kirschner wire fixation. The dorsal approach is often favored in cases with perilunate dislocations for ligament repair and stabilization, while volar approaches may be used for DRFs [5,11]. There was also a need for dual approaches, dorsal and volar, as seen in complex injuries [8].

There may be several limitations related to the rare association observed between a dorsal perilunate dislocation and DRF. The individual presentation of a dorsal perilunate dislocation in the emergency department can be a frequently missed diagnosis. Pelrine et al. detail how approximately 25% of perilunate dislocations are missed on initial assessment of the injury in the emergency department [15]. This can potentially affect the long-term results of this injury. This article further examined the impact of missed perilunate dislocation diagnoses on the treatment and outcomes of this injury. Similarly, Herzberg et al. conducted a retrospective study noting missed initial diagnoses in 41 of 166 perilunate dislocations and fracture-dislocations [16]. A limitation of our study is the short follow-up period. The combination of a DRF and perilunate dislocation would need further follow-up to evaluate for late complications such as stiffness, instability, arthritis, and avascular necrosis. Long-term clinical DASH scores, functional outcomes, and radiographic assessment could not be provided because of the short follow-up period.

## Conclusions

This case details the initial presentation, diagnosis, and surgical treatment of the combined injuries of a dorsal perilunate dislocation and a comminuted intra-articular fracture of the distal radius. DRFs are common injuries that physicians encounter in the emergency room. However, their coexistence with perilunate dislocations is extremely rare, with only a few cases being reported in the literature; thus, heightened clinical suspicion is essential during initial assessment of high-energy wrist trauma.

This case adds to the limited body of literature on combined high-energy carpal and distal radius injuries and highlights the importance of a dual-approach surgical strategy, early recognition, and early reduction with internal fixation when approaching these injuries. The rare complex nature of the combined dorsal perilunate dislocation and DRF presentation will help further enhance the proficiency of emergency room physicians and surgeons. Long-term follow-up is critical in these injuries to identify potential complications.
